# American Dental Association

**Published:** 1893-02

**Authors:** 


### Reports of Society Meetings.

### AMERICAN DENTAL ASSOCIATION.

*Second Day.—Evening Session.*

(Continued from page 60.)

### DISCUSSION ON DR. MORRISON'S AND DR. CRAVENS'S PAPERS JOINTLY.

*Dr. Head.*—I should like to ask if Dr. Morrison will give us some idea of how many of his implanted teeth have lasted five years after the operation has been completed?

*Dr. Morrison.*—I should judge not more than twenty per cent. I have a list, but I have not looked at it for some time.

*Dr. Swasey.*—On this subject of implantation, I was impressed with the fact that Dr. Morrison failed to secure a favorable result with his first case. In answer to the query how long the teeth lasted, he said that about twenty per cent. of his cases have been successful for five years. I had a call from Dr. Younger some time ago, and I felt that I had been remiss in the practice of dentistry from the eloquence with which he described his operations.

On this subject of matrices, it seemed to me that he objected to anything malleable in approximal cavities. I would not use a steel matrix, but should want something unyielding. I prefer a brass one that can be hammered on to the wall of the approximal cavities. He also spoke of the obscure walls of the cavity. I do not believe many men fill cavities having obscure walls. His meaning is not clear. I would not fill a cavity that I could not see. When this is done or the dentist fails to separate the teeth and uses a matrix that cannot be wedged on the other teeth, so that he cannot impact every portion of the tooth, I should infer he was not skilled in operative dentistry.

This question of matrices has been a great boon for many people, but it is not for one who prefers to fill a tooth right, who is careful that he gets his filling to the cervical margin; the only way to do it is to feel that when the matrix and the rubber dam are adjusted, and the cavity dry, he has a simple cavity. There is the secret of the adjustment of the matrix.

*Dr. McKellops.*—I have a little to say on the subject of the use of the matrix, as I think it a very important instrument. Dr. Jack did the profession a great service when he brought out this instrument. In the first place, it gives plenty of room. I must have room to see what I am doing. In that paper the author says he cannot bring up the edges. I bevel the edges so that I know they will be perfect when I finish the plug. I take a matrix, and I fit it. If I find the neck of the tooth a little too small, I place on my jack-screws or Perry's separator to hold it in position. I get my rubber dam on first, and then put those in to hold the tooth firm, and it gives no pain to the patient. If it is properly done the patient never feels the effect of the blow of the mallet. When you do away with that you cause suffering; and it should be the study of the artist to find out how much relief he can give his patient in operating.

In placing a matrix on, at the base of the gum it stands a little open. I want something to hold the matrix to the wall. I am going to fill the anterior cavity. I take a piece of orange-wood, and I bevel it the shape needed. Then a little varnish is added and it is forced in. That holds the matrix in place, and I am ready to go to work. I can spend two or three hours on the tooth without hurting the patient. It is a good thing when it is properly used, and the spaces are necessary.

As far as the implantation of teeth is concerned, we have all seen the operation. I have had a great deal to do with Dr. Younger, and have seen many of his cases. Some have been successful, but the majority have been complete failures. A few last five or six years, but that is all. I am not in favor of extracting teeth, but do sometimes. I will go to any extreme, and will go anywhere, to see a man operate. If he has anything new, I want it, to give to the profession, and to afford my patients relief and comfort.

*Dr. Taft.*—This seems to be a small matter, but after all it is a very important one. There is quite a diversity of opinion in regard to the use of this appliance—the matrix—for filling teeth. Some use it a great deal, others very little; some do not use it at all. A variety of appliances of this kind have been suggested to the profession. Dr. Jack many years ago brought out his matrix, a very good instrument or appliance, properly constructed, made of steel thin as possible, concave on the side that came next to the cavity, which was a proper form, of course. Then followed thin pieces of steel or other metals, placed simply in the space forming a simple cavity. Then there is the band matrix, which is made in various forms. Brophy has a band matrix, and also many others have introduced the same principle. The German silver band matrix referred to was suggested by Dr. Herbst some years ago, when he was in this country,—taking a thin piece of German silver, placing it about the tooth, and with forceps drawing the ends together, fitting it closely on the tooth, and then soldering it. It was a simple method of making a good band for some cases. Now, all these appliances may be good sometimes, but we find a great deal of faulty work resulting in operations made with this instrument. It is often observed that there are defective borders on the fillings; it may have come squarely against the edge of the border, and it will be found impossible to make a perfect adaptation of the filling at that part of the tooth.

While all the forms of matrices that have been suggested are valuable for certain cases, all these varieties should be at hand. Some will be better adapted to one case than to another, and so it will be all through the list. One point that has not been alluded to is the use of the matrix that will come up one-fourth, one-third, or one-half upon the cavity, as you choose. This forms the lower portion of the cavity, and it can be perfectly filled with ordinary care. After the upper part of the filling has been placed in position the matrix may be removed, and the remaining portion of the filling made without the matrix, care being taken, in building up along the lateral borders, to have it perfectly adapted and sufficiently flush or full to make a complete finish to the filling. In that way the difficulty that sometimes occurs in the use of the matrix when it extends to the grinding-surface of the tooth is avoided, and a good filling may be made, whereas if the full matrix were used, the border would be more or less defective. In that way there is room for the entrance of the instrument, the file or disk, the corrundum wheel, or whatever is used to finish off. In many instances I find this method of procedure better than using a band or ordinary matrix, and the difficulty that is liable to occur is avoided.

*Dr. Darby.*—I do not know how much those before me have said on implantation, as I have just entered the room; but it seems to me the subject is worthy of attention. There is a difference of opinion in regard to the value of this operation. The reply to the question as to how long these operations last would lead many to believe that they last but a short time. I do not pretend to say that implanted teeth will be permanent, but many teeth that I have seen have done good service for six or seven years, and are good to-day. I may be justified in relating a case which came under my observation within the past year. A lady went to San Francisco and had six teeth implanted by Dr. Younger,—four cuspids and two central incisors. For six years these teeth did good service. An accidental bite, as she termed it, had loosened and caused one of the central incisors to fall out. The lady applied to me for treatment, and I suggested that she allow the socket to heal, the root having absorbed. I introduced a little denture with a single tooth on it. A few months afterwards I made a new socket through dense bone, and I happened to have in my collection a tooth nicely adapted, and well matching the other incisor. This tooth was implanted, and after a few months it became firm, and is so to-day. For a period of seven years at least that central incisor and four bicuspids have been doing good work. Whether they will remain so, I cannot say. I am of the opinion that the majority of these teeth will be failures. I do not think we always distinguish between the two kinds of attachment. I allude now to those cases where we have bony ankylosis. In such I think the tooth will be permanent. My own experience has not been equal to that of many other gentlemen. I have implanted thirty-six teeth, and, as far as I know, but five have been failures. Some have been in between three and four years, and to all intents and purposes are as good as any in the mouth. I can recall many instances in which these teeth are perfectly firm, and I should not know that they had been implanted teeth if I had not implanted them myself. Some cases are exceedingly valuable. Take a central incisor, for instance; no other tooth in the mouth has been lost; from accident, breaking off of the crown, or any other reason, it becomes desirable to implant a tooth. In many instances it may last three or four years, and if it is lost, another can be implanted. In some instances I have had central or lateral incisors that had been crowned and the roots split. I have had good roots upon teeth that had defective crowns. I have crowned an artificial tooth on a good root, and it has taken hold as well as a whole natural tooth. In instances of that kind I think implantation is extremely desirable, and in the event of failure we can implant another.

I will relate a case of a young lady. She had lost two teeth, and was wearing a gold-plate. After she was married, her husband brought her to me, and I found she had lost the two bicuspids in the superior jaw. It was then that Dr. Younger first suggested the operation of implantation. I had seen him perform the operation, and I suggested implanting one bicuspid. I had one that I had drawn from the mouth of a child in correcting irregularity. The lady sailed for Europe, and she wrote me that the tooth was firmly fixed, and when she came back she wanted the other one implanted. I did so, and she went to Europe again, and when she returned, after two years, both teeth were as solid as the day I put them in. Even if those teeth failed in five years or more, it would be easy to implant others. I do not want the operation of implantation to receive any discouragement from those who have spoken on the subject.

*Dr. Morgan.*—There have been some views advanced that I cannot let pass unchallenged. I agree with most that has been said. The first criticism that I will make, however, is on what Dr. Darby has said about ankylosis. I do not understand that there is any such thing as bony ankylosis. We have ankylosis of the parts, but it is a union of the cartilage, not of the bone. Taking that view of it, I think bony ankylosis is an improper term to apply. I think it becomes encysted, and the fibrous substances hold it in place, but there is never any bony union; the dead and the living do not unite, and when you have a tooth out of the mouth for eight or ten years, it is dead in the full sense of the word.

Two gentlemen have said that teeth cannot be filled unless you can look into the cavity and see every portion of it. Some of my best work has been done where I could only see a small portion, and I have been in the habit of doing it, as my patients and my brother practitioners say, successfully ever since I commenced to practise. Take, for instance, the posterior surface of a second or third inferior molar, which occasionally requires filling. I have often filled one where only a small portion of the pulp could be seen. Of course, I use a glass to aid me, but I have always relied as much on my sense of touch as I did upon the eye or the glass. Take the left inferior maxilla. I stand on the left side of my patient, and with an instrument carry into the cavity, for the beginning, a cylinder of soft or non-cohesive gold, and then I fill it until it is full. I do the packing by the sense of touch, and then compress the surface and polish it, relying upon the glass to secure the finish I desire. I have done it for nearly fifty years, and I know it has been successful, because of the results. I always begin with the easiest mode of filling cavities, and for that purpose I use non-cohesive gold. I do it satisfactorily without looking into the cavity. I know that one gentleman in this room has been in the habit of doing that thing for forty years, and he could tell exactly the same story. I will also say that while I have used cohesive gold almost from its first introduction as a material for filling teeth, I still cling to my first love to a great extent, and use quite a large proportion of non-cohesive gold.

*Dr. McKellops.*—If pure gold is not adhesive, what is it? If it is pure, it will adhere. You cannot get over it, call it what you will. As far as seeing into a cavity is concerned, I say you cannot take a mirror into a cavity. It cannot be prepared right. If it is an inferior wisdom-tooth, it cannot be filled with the success that I can fill it by going down and seeing what I am doing. It cannot be a success, and I challenge it.

*Dr. Morgan.*—I do not claim to make perfect fillings. I claim to make such as preserve the teeth, and I have yet to see operations from any gentleman in this country whose fillings are perfect when brought to the proper test.

*Dr. Ottofy.*—I was very much delighted in listening to the remarks of Dr. Darby on implantation. I was rather diffident about the subject at first, but I am at present as much of an enthusiast as I ever was. I think when Dr. Younger came East he was obliged to implant teeth indiscriminately in mouths not properly suited for it. Unfortunately, under those circumstances, nothing else could happen but what did, and that was that most of the teeth failed. There is no question that his private practice is entirely different from that. I agree with almost everything that Dr. Darby has said, but there are very few perfect natural crowns to be had, and often those that seem perfect have the enamel chipped, and I have lost several beautiful cases from the fact that the enamel and portion of the tooth have broken away beyond the alveolar border, causing irritation and eventually loss of the tooth. At the present time I invariably cut off the crown and attach a Logan crown, making the connection between the Logan crown and the root of gold, simply attaching the Logan crown carelessly, so as to leave a space where I put in a gold filling. In that way the tooth is perfectly strong, and can be changed in shape if desired. I have some cases that are from five to six years old, and there is nothing more beautiful than an implanted tooth in a perfect condition. Most of my first cases failed. They were poorly done, and they were not suitable for the operation. There is hardly any case that I have selected for implantation that is not in good condition, and they range from two to five years. Some one has asked, How long will they last? Dr. Younger first implanted those teeth about seven years ago. Much of the work has been done within the past five years, and we can safely say that they will last from four to seven years. Others can be implanted when these are lost. In cases where a root absolutely must come out, the socket can be kept, and a tooth implanted without any pain to the patient.

*Dr. Jackson.*—I would request that Dr. Allport give his experience in filling teeth with soft gold. Many of the younger members would be greatly benefited by Dr. Allport's experience. Many of us have lost sight of the discussions which have taken place on the subject. I think soft gold is considered a good safe filling. Why should we not be put in line again to study out and know the advantages in the use of this material?

*Dr. Allport.*—In regard to perfect fillings, I want to say that a perfect filling would be one so dense that it would be like molten gold, and with every portion of that cavity filled as closely as a most perfect-fitting cork could fill it. However, such a filling was never put in by any living man. A saving filling is what we need; of course we want perfect fillings, but we cannot get them. Dr. Jackson spoke of the use of non-cohesive or soft gold. I suppose he means what I regard as the relative value of the two kinds of gold, cohesive and non-cohesive. Cohesive is good in some ways and non-cohesive is good in others. Neither will perfectly take the place of the other. In our present practice we could not do justice to our patients without using both. History not only teaches us of the past, but throws light on the future, and there is nothing better than to look back on the development of dental operations to get at the relative values of these two golds. Let us go back forty years or more. What had we then? But one kind of gold, and that was non-cohesive. At that time it seldom happened that a filling put in by a good operator discolored. Frequently we found them soft after they were taken out, and yet they retained their color. The trouble was that a portion of the fillings would be lost. They were put in by lateral pressure, and by forcing one piece into another, and when failure took place it did so by one piece of the filling coming off and becoming separated from the other pieces. Half would come out, and yet the balance would be perfectly good, and I have even repaired such fillings. What did we need then? We required something to make a good surface to that filling and prevent pieces from falling off. Then came crystal or sponge gold. All crystal or sponge gold is cohesive, and this gold was very useful to be used over non-cohesive gold. It was exactly what we wanted at that time. The fillings of Dr. Morgan and Dr. Westcott did not discolor. They introduced this crystal gold, and everybody used it. What was the result? Very soon we heard that the appearance of teeth was so marked that our journals were filled with accounts of it. The gold discolored, and it was said it was because the gold was impure. But that was not so; it discolored because of leakage; exactly the same as they do to-day, because they do not fit the walls of the cavity. Take Dunning's or Townsend's or Harwood's, and you find no better fillings. There we have the two filling-materials, the non-cohesive and the other, which we need to put there as an enamel, and the sooner we come to it the sooner we will get to a rational, sensible practice.

The trouble is, we have lost our old way of operating. It has been said that the best way is to see the cavity. I have filled hundreds of teeth that I did not see, except with the glass. With the fingers properly educated, as Dr. Morgan said, with eyes at the end of the instrument, a great deal can be done. I use mallet force every day, but I cannot do with the mallet what I can do with my hand. I have seen hundreds of old fillings, that have been put in between approximate teeth with old-fashioned instruments, that are perfectly good to-day. You cannot do it with your mallet pluggers and your cohesive gold. They would destroy a large portion of the teeth that would not be destroyed if you used the old-fashioned non-cohesive gold. Suppose you want to fill the edge of a posterior surface, and there is an undercut. You cannot do it with the plugger, but with the old instrument you can do it. You have lost more than you can imagine in not knowing the old-fashioned way of operating. I think as much of our colleges as any one, but while they pretend to teach the old-fashioned ways, they do not do so. The manipulation is so different, that a man who graduates does not know how to use non-cohesive gold. The old way was to take the gold and crowd it laterally towards the wall and bring it in front, and then in the centre, and put it all over. Now that they have the cohesive gold, they say everything must be driven from the bottom up, or from the top down, all over the floor of the cavity. You can draw a sled up-hill, but you can slide it down much better. You try your instrument, and the tendency every time is to draw it from the wall, and the only way you can avoid it is to make the centre of the cavity higher. Suppose you watch every time you force your instrument in; you will see that the tendency is to press it exactly where you want it,—where your cavity leaks. You can do many difficult things. A man with much skill and time can put in a good filling with cohesive gold, but a man with much less skill and time can put in just as good a one with non-cohesive gold.

*Dr. Swasey.*—If I understood the sense of the paper on which I made the remarks, it was that in filling teeth with a matrix, a portion of the cavity was not visible. Dr. Morgan and Dr. Allport have endeavored to convince us that they have filled a tooth that they never saw, except by touch and with a glass. I have seen things through a glass which appeared more vivid than with the naked eye. I have filled a tooth with non-cohesive gold without seeing the cavity. I do not think that these men fill cavities oftener than myself, although they are older; but when they insinuate that every portion of the cavity must be seen, it was a misapprehension of my remarks. I want you to understand that I feel that there is a place for soft foil that has been established many years ago. As Dr. Allport said, the addition of cohesive foil makes it a perfect filling, but if one tells me that he fills a cavity without seeing the walls, I should be very doubtful as to the result.

*Dr. Watkins.*—I was much interested in the remarks on this paper on implantation. I had commenced to think that the subject had died away, but to-night we have had a very excellent discussion, and a very instructive one, in regard to it. I have performed some implantation, but not as much as others who have spoken. I want to relate just one case which I implanted five years ago. After it had been in the mouth for four years, perfectly solid, the outer cusp was split off. I removed the inner cusp and crowned the tooth. That was a year ago, and it is in good condition now.

*Dr. Brophy.*—Many years ago, when I was looking up the history of this matter, I found in the *American Journal of Dental Science* an article which appeared about thirty-five years ago from the pen of our esteemed friend, Dr. Dwinelle, on the use of the matrix, where he first proposed the use of this device, and I desire that our record should show this fact. The matrix is a most excellent device when properly understood and used, but when it is not understood and not properly used, it ought to be left entirely alone.

The Secretary then announced that the committee of three recommended in the president's address will consist of Drs. E. C. Kirk, Louis Jack, and J. N. Crouse.

Adjourned.

